# Gene coexpression network analysis reveals perirenal adipose tissue as an important target of prenatal malnutrition in sheep

**DOI:** 10.1152/physiolgenomics.00128.2022

**Published:** 2023-07-17

**Authors:** Sharmila Ahmad, Markus Hodal Drag, Suraya Mohamad Salleh, Zexi Cai, Mette Olaf Nielsen

**Affiliations:** ^1^Department of Animal Science, Faculty of Agriculture, Universiti Putra Malaysia, Serdang, Selangor, Malaysia; ^2^Research Unit of Nutrition, Department of Animal and Veterinary Sciences, Aarhus University, Tjele, Denmark; ^3^Novo Nordisk Foundation Center for Basic Metabolic Research, Faculty of Health and Medical Sciences, University of Copenhagen, Copenhagen, Denmark; ^4^Copenhagen Zoo, Frederiksberg, Denmark; ^5^Department of Animal Nutrition and Management, Swedish University of Agricultural Sciences, Uppsala, Sweden; ^6^Centre for Quantitative Genetics and Genomics, Aarhus University, Tjele, Denmark

**Keywords:** early postnatal malnutrition, perirenal adipose tissue, prenatal malnutrition, subcutaneous adipose tissue, WGCNA

## Abstract

We have previously demonstrated that pre- and early postnatal malnutrition in sheep induced depot- and sex-specific changes in adipose morphological features, metabolic outcomes, and transcriptome in adulthood, with perirenal (PER) as the major target followed by subcutaneous (SUB) adipose tissue. We aimed to identify coexpressed and hub genes in SUB and PER to identify the underlying molecular mechanisms contributing to the early nutritional programming of adipose-related phenotypic outcomes. Transcriptomes of SUB and PER of male and female adult sheep with different pre- and early postnatal nutrition histories were used to construct networks of coexpressed genes likely to be functionally associated with pre- and early postnatal nutrition histories and phenotypic traits using weighted gene coexpression network analysis. The modules from PER showed enrichment of cell cycle regulation, gene expression, transmembrane transport, and metabolic processes associated with both sexes’ prenatal nutrition. In SUB (only males), a module of enriched adenosine diphosphate metabolism and development correlated with prenatal nutrition. Sex-specific module enrichments were found in PER, such as chromatin modification in the male network but histone modification and mitochondria- and oxidative phosphorylation-related functions in the female network. These sex-specific modules correlated with prenatal nutrition and adipocyte size distribution patterns. Our results point to PER as a primary target of prenatal malnutrition compared to SUB, which played only a minor role. The prenatal programming of gene expression and cell cycle, potentially through epigenetic modifications, might be underlying mechanisms responsible for observed changes in PER expandability and adipocyte-size distribution patterns in adulthood in both sexes.

## INTRODUCTION

Adipose tissue functions as an energy reservoir while also playing an essential role in the development of various metabolic diseases, such as type II diabetes, insulin resistance, and cardiovascular disorders (1). In humans, subcutaneous adipose tissue (SUB) is associated with obesity/adiposity-related complications such as diabetes (2, 3), whereas perirenal adipose tissue (PER) thickness is correlated with kidney health and cardiometabolic risk factors (4–6). Large animal models such as sheep have previously been used to investigate the interplay between insulin sensitivity, type II diabetes, and metabolic response to exercise and adiposity (7–9). Similarly, using the Copenhagen sheep model, we have previously demonstrated significant changes in the PER transcriptome of 2.5-yr-old adults exposed to prenatal undernutrition (LOW) followed by a mismatching obesogenic, high-carbohydrate-high-fat (HCHF) diet during the first 6 mo of life (adolescent lambs) (10). In these nutritionally mismatched LOW-HCHF adult sheep, the affected molecular pathways included cholesterol metabolism, whereas early postnatal HCHF feeding targeted biological processes related to kidney cell differentiation. These findings are interesting since kidney weight was reduced by one-third in adolescent LOW-HCHF lambs associated with a massive expansion of PER mass, and in LOW-HCHF sheep, hypercholesterolemia even persisted into adulthood after 2 yr of dietary correction (10–12). Altogether, the findings support the idea that SUB and PER adipose tissues play crucial but distinct roles concerning the long-term risk of metabolic complications, where SUB is considered a more “healthy” fat depot (13, 14).

Adipose tissue comprises various cells, including adipocytes, that vary in size. The relationship between different adipose cell sizes and metabolic diseases such as insulin resistance and diabetes has been extensively studied (15–19). Nevertheless, it is still hard to define the exact size threshold or the proportion of various adipocyte sizes (i.e., small vs. large) that can be associated with impaired adipose-related metabolic disorders (20). In the case of obesity, the inability of adipose precursor cells to differentiate into mature cells, the ability of small fat cells to expand, and the failure of large fat cells to enlarge can lead to an impaired ability of adipose tissue to store excess energy, which consequently increases free fatty acids and adipokines that can further cause disturbed systemic metabolism (20). Studies in the rat have demonstrated that elevated plasma triglyceride concentrations and larger adipocyte size in the SUB and retroperitoneal fat depots were observed in the prenatally undernourished adult male rat, suggesting the presence of significant long-term metabolic programming of adipose tissue physiology in response to prenatal undernutrition (21).

Previous studies have also reported that in utero undernutrition alters maternal-to-fetal hormone messengers such as growth hormone, insulin, and thyroid, all closely related to fetal growth and development (22). The thyroid hormone is crucial for adipose development and functions (23–25), and previous studies have shown that in utero malnutrition in humans (26), sheep (27), and rats (28) permanently altered the thyroid function and hypothalamus-pituitary-thyroid axis.

Similarly, using our Copenhagen sheep model of malnutrition, we have studied and observed the long-term impacts of late gestation under- and overnutrition on various traits such as histomorphometric changes of SUB and PER adipose tissue (29), changes in plasma metabolites levels of glucose, nonesterified fatty acids (NEFA), triglycerides (TG), blood urea nitrogen, creatinine, lactate, β-hydroxybutyrate (BOHB), γ-glutamyl transferase (GGT), and cholesterol levels (12), serum hormones level of thyroid-stimulating hormone (TSH), triiodothyronine (T_3_), and thyroxine (T_4_) (27) in 2.5-yr-old sheep measured during so-called intravenous tolerance tests. By 2.5 yr of age, we observed that in PER (not SUB), male sheep (not female) that had been exposed to late gestation undernutrition had reduced adipocyte hypertrophic ability, whereas male sheep exposed to prenatal overnutrition had increased PER hypertrophic expandability more similar to what was observed in all females (exposed and nonexposed to prenatal malnutrition) (29). In addition, we found that LOW-HCHF adult sheep (male and female) were consistently hypercholesterolemic, hyperurecemic, and hypercreatinemic compared to all other groups (12, 30), and baseline plasma levels (before bolus injections in tolerance tests) of NEFA and TG were also highest in LOW-HCHF sheep.

The transcriptome is the entire set of messenger RNA (mRNA) expressed by a cell or tissue of an organism of a given genotype under internal or external influences (31). The method of transcriptomic analysis in understanding the functional elements of the genome includes differentially expressed genes (DEGs) and weighted gene coexpression network analysis (WGCNA). The latter has many advantages since it also allows evaluating the association of modules (coregulated genes) with phenotypic sample traits using network properties (31). We have previously performed the DEGs analysis of adipose tissue (SUB and PER) of sheep with different histories of early life malnutrition (10), and in this study, we further perform the WGCNA as it is now widely used for extracting information (e.g., genes and their biological functions and pathways) from large transcriptome data sets, hence allowing the identification of coregulated genes, candidate biomarker genes, biological processes, and pathways associated with particular phenotypes or clinical variables of interest (32–35). Specifically, WGCNA has been used previously to improve our understanding of the molecular pathways underpinning the role of adipose tissues concerning various conditions (e.g., obesity, exercise, type II diabetes) and their associated health implications in both human and animal models (2, 36, 37).

In this study, we aimed to characterize module-trait relationships in SUB and PER obtained from 2.5-yr-old male (♂) and female (♀) sheep that had been exposed to different combinations of prenatal nutrition during late gestation (according to requirements: NORM, undernutrition: LOW, or overnutrition: HIGH) and during early postnatal life (until 6 mo of life, i.e., after puberty; moderate diet: CONV or obesogenic diet: HCHF). To compare the expression of modules across the conditions [i.e., sheep with different prenatal (NORM, LOW, HIGH) and postnatal (CONV, HCHF) nutrition histories], the comparison was made within each depot and sexes separately, giving rise to four gene networks namely, SUB♂, SUB♀, PER♂, and PER♀. Studies have shown when analyzing the molecular responses of adipose tissue to diet, the anatomical location is an important factor to consider since there are differences in adipogenic factors, responses to hormones, metabolic properties, or secretion of inflammatory cytokines that have been observed between SUB and visceral fats (38). Similarly, using machine learning algorithms followed by WGCNA, each adipose depot can be segregated into unique and concise modules containing coexpressed genes involved in the adipose function (39). In addition, the unsupervised hierarchical clustering and principle component analysis revealed that each depot forms a distinct cluster, suggesting a strong fit between the computational model and the existence of intrinsic differences in gene expression between depots (39). Besides the anatomical location, a study showed that to best estimate the best associations between modules of phenotypes of interest, sexes were analyzed separately since the comparison between the sexes is complicated because sex bias in networks can be tissue specific (40).

The depot- and sex-specific gene network approach was anticipated to help us discover molecular signaling pathways and hub genes associated with the observed changes in adipose morphological traits and other phenotypic manifestations in adulthood. Hub gene(s) centrally placed in a gene network usually play(s) a vital role in regulating the expression of many genes as determined by their connectivity with these other genes. Our hypotheses were *1*) tissue- and sex-specific changes in gene coexpression networks, particularly in PER, are associated with adipose-related metabolic disturbances induced by pre- and postnatal nutrition, and *2*) a WGCNA approach can identify potential genetic biomarkers responsible for such metabolic alteration.

## MATERIALS AND METHODS

### Ethics Statement

The animal trial was conducted at an experimental facility in Rosenlund, Lynge, Denmark, under the supervision of the Faculty of Health and Medical Sciences, University of Copenhagen, Denmark. The National Committee on Animal Experimentation, Denmark, approved all experimental animal procedures (License No. 2010/561-1853).

### Experimental Design, Diets, and Adipose Tissue Samples Collection

The experimental procedures and diets have previously been reported (10–12). The experiment was a 3 × 2 factorial design, in which 36 twin-bearing crossbred Texel ewes in the last 6 weeks of gestation (term: ∼147 days) were assigned to either NORM (fulfilling the energy and protein requirement according to Danish feeding standards), LOW (50% of NORM), or HIGH (150% and 110% of daily energy and protein requirements, respectively) diets. After suckling their dams, the first 3 days after birth, the two twin lambs were assigned to separate diets until 6 mo of age (after puberty). They were artificially reared on a moderate conventional (CONV) or high-carbohydrate-high-fat (HCHF) diet. The CONV diet comprised restricted supplementation with high-quality hay and milk replacer during the first 8 wk of life, whereby moderate growth rates of ∼225 g·day^−1^ were achieved. The HCHF diet consisted of a mix of 37% fat dairy cream with milk replacer in a 1:1 ratio (maximum: 2.5 L·day^−1^) supplemented with rolled maize (maximum: 2 kg·day^−1^) and a small amount of barley straw. At 6 mo of age, subgroups of lambs from each of the six treatment groups were euthanized. The remaining sheep, which were used in this part of the study, were managed in two sex-divided groups and fed the same low-fat hay-based diet ad libitum supplemented with barley until 1 yr of age (adjusted to achieve a moderate and consistent growth rate of ∼225 g·day^−1^ until 1 yr of age) and ad libitum with hay only until 2.5 yr of age. The sheep were euthanized at 2.5 yr of age (adulthood) by decapitation after induction of general anesthesia by intravenous administration of 5–6 mg kg/body weight of propofol (B. Braun, Melsungen, Germany). At autopsy, specimens of SUB and PER were dissected and immediately stored in RNAlater (RNAlater@Solution, Ambion, The RNA Company) for 24 h and then transferred into empty tubes and stored at −80°C pending analysis for the present part of the study. Thirty-one (14 males: 17 females) and 36 (17 males: 19 females) tissue samples of SUB and PER, respectively, were used for the RNA-sequencing analyses.

### Phenotypic Traits Used in Module-Traits Relationships Analyses

All data for phenotypic traits used in this study were derived from previous reports based on the same sheep from the same experiment (11, 12, 29, 30, 41, 42). The total weights of SUB, PER, and other internal organs (kidney, liver, heart, adrenal glands) were recorded at autopsy at 2.5 yr of age (42). The histomorphometric characteristics of SUB and PER [e.g., average cross-sectional area (CSA) of adipocytes, arbitrary cell-number index (CNI) to indicate total numbers of adipocytes in SUB and PER, adipocyte size distribution (categorized into cell size classes of 0–40, 40–200, 200–400, 400–800, 800–1,600, 1,600–3,200, 3,200–6,400, 6,400–12,800, 12,800–25,600, and 25,600–36,000 µm^2^)] were derived from histomorphometric analyses previously described in detail (29, 42). Plasma metabolite levels ([glucose, nonesterified fatty acids (NEFA), triglycerides (TG), blood urea nitrogen, creatinine, lactate, β-hydroxybutyrate (BOHB), γ-glutamyl transferase (GGT)] and cholesterol levels were measured during so-called intravenous tolerance tests, i.e., before and after intravenous bolus injections of glucose (GTT), insulin (ITT), and propionate (the latter test conducted both during the fed state: PTT_fed; and fasted state: PTT_fast). These tolerance tests were performed with the adult sheep during the last weeks preceding the autopsy (see Ref. 12 for details). The serum hormones level of thyroid-stimulating hormone (TSH), triiodothyronine (T_3_), and thyroxine (T_4_) were determined in the 2.5-yr-old sheep during an intravenous thyroxine tolerance test (iTTT) conducted over 2 days following overnight fasting (27).

### The RNA Extraction, Library Preparation, RNA Sequencing, Data Preprocessing, and Weighted Gene Coexpression Network Construction

Details on total RNA extraction, library construction, sequencing, and data preprocessing have been reported previously (10). In brief, the RNA of 31 (14 males: 17 females) and 36 (17 males: 19 females) of SUB and PER tissue, respectively, were extracted and sequenced by Novogene (HK) Company Limited, Hong Kong. The steps for the total RNA extraction and quality control included the following: *1*) total RNA extraction following the TRizol protocol and removal of DNA using DNAase; *2*) total RNA quality and RNA degradation and potential contamination quantification using Nanodrop (Thermo Fisher Scientific, Carlsbad, CA), followed by agarose gel electrophoresis, respectively; *3*) total RNA quantity and integrity assessment using the Agilent 2100 Bioanalyzer (Agilent Technologies, Santa Clara, CA); and *4*) total RNA concentration measurement using the Qubit RNA Assay Kit and a Qubit 2.0 Fluorometer (Life Technologies, Carlsbad, CA).

Following the RNA quality control, the library cDNA libraries were prepared. First, the ribosomal RNAs (rRNAs) were removed using the Ribo-Zero RNA Removal Kit (EPICENTRE Biotechnologies, Madison, WI). After that, the fragmentation buffer was used to randomly fragment the purified RNA into short fragments of 250 and 300 bp. The random hexamer primers (Illumina) and dNTPs (dUTP, dATP, dGTP, and dCTP) were then added to synthesize the first-strand cDNA, followed by DNA polY-A tail ligation to the sequencing joint. The AMPure XP beads were used to purify the correct-sized fragments. The USER Enzyme was used to degrade the cDNA strands containing U instead of T (USER Enzyme; BioLabs Inc, UK), and the first-strand cDNA was sequenced, thereby preserving the direction of the RNA. Finally, PCR amplification was conducted, and the products were purified (AMPure XP beads) for constructing the cDNA libraries. The latter’s quality was assessed using the Agilent BioAnalyzer 2100 system (Agilent Technologies, Santa Clara, CA) and quantitative PCR. The libraries were sequenced on an illumine Hiseq 4000 platform, and 150-bp-long paired-end reads were generated.

Before WGCNA construction, the base-paired end reads were preprocessed. The quality of pair-end reads of all samples was examined using the FastQC v0.11.18 software (43). After that, the low-quality reads and adaptor sequences were removed using Trimomatic v0.39 software (44). The rRNA contamination was removed using a database from SILVA v132 (45). The RNA sequences were retrieved from SILVA and processed to build a bwa index file using Burrows-Wheeler-Aligner (BWA) v0.7.17-r1188 software (46). The clean data were mapped to the SILVA database using bwa mem, and then the paired and unmapped sequences were extracted using Samtools v1.10 (47) and Bedtools v2.29.2 (48). The generated “clean reads” were used for all downstream bioinformatics analyses.

The mean numbers of clean reads per sample obtained from SUB and PER were 34,137,165 and 34,818,481 (10). The STAR c2.7.3a was used to map the clean reads to the reference genome Oar v3.1.99 gtf (49) with a default parameter, except that a maximum of eight mismatches were allowed (50). The quality control was performed using Qualimap v2.2 (51). After mapping, each SMA file obtained was sorted and transformed into a file by Samtools v1.9 (47), and the gene expression counts were computed using HTSeq v0.11.8 (52).

Before gene network constructions, genes with less than one count per million were removed (53). Then, the gene expressions were normalized using the default parameter from DESeq2 package v1.26.0 (54) in RStudio (v1.2.5042) (55) (correcting for the library size and RNA composition biases) and fitted with a generalized linear model assuming a negative binomial distribution of the read counts (54) following this model:

$$Y_{\text{ij}}=∼1$$

where *Y*_ij_ is the gene expression count for gene *i* in sample *j* ∼1 negative binomial regression.

The normalized data were then log2 transformed following the WGCNA recommendation and used to construct the coexpression gene network using the WGCNA package (56) in R studio (55). The unsigned network type was used to build the gene coexpression network. First, sample clustering was performed, in which samples were clustered based on the expression of all genes, and any obvious sample outliers were removed (57). We used hierarchical clustering to produce a hierarchical clustering tree (dendrogram) of genes. In total, 1 out of 14 animals in the SUB♂ data set, while 2 out of 17, 14, and 17 animals in the SUB♀, PER♂, and PER♀ data set, respectively, were detected and removed from further analyses. Next, the soft threshold power (B) was fixed to 7, 5, 12, and 9 for SUB♂, SUB♀, PER♂, and PER♀ datasets, respectively, after applying the function of pickSoftThreshold to reach a scale-free topology index (*R*^2^) above 0.8. Afterward, the topological overlap matrix was computed from the adjacency matrix containing the pairwise Pearson correlation coefficients. Next, the modules of coexpressed genes were identified and merged using the dynamic tree-cut algorithm. Each module was assigned a unique color to distinguish among the modules found. Next, the module eigengene (ME) was computed for each module, and Pearson’s correlations were calculated between each ME and each trait to estimate module-trait relationships (MTRs). Finally, the modules of interest were selected based on the MTR with a false discovery rate (FDR) value of *P* < 0.05 considered significant.

### Functional Enrichment Analysis

The functional enrichment analysis [Gene Ontology (GO) terms (i.e., Biological Processes, Cellular Component, and Molecular Function) and Kyoto Encyclopedia of Genes and Genomes (KEGG) pathways] of modules showing significant correlation with the studied traits was performed using the Cytoscape v.3.8.2 plug-in ClueGO (58, 59), following our previous publication (10). The lists of genes within each of the selected modules were uploaded in the application, and Ovis aries (Taxonomy ID: 9940) was used as the reference genome to find significant enrichments. The selection criteria for the enrichments were based on a two-sided hypergeometric test with Benjamini-Hochberg corrected *P* < 0.05. Other criteria include a minimum of 3–20 GO term levels and a minimum number of 3 genes or at least 4% genes in the respective terms.

### Identification of Hub Genes (via Protein-Protein Interaction Networks)

Hub genes were selected based on the module membership (MM) (56, 60) and using the CytoHubba application (61). First, hub genes were selected from each module based on MM, representing the degree to which a gene is correlated with the module’s eigengene. The MM is strongly correlated with intramodular connectivity and can therefore be used to reveal master regulatory genes or hub genes, as regulating the expression of other genes in the module can prioritize potential candidate genes (53, 62). Genes with the highest MM (MM ≥0.8 and *P* < 0.05) in each selected module that significantly correlated to at least one trait of interest were prioritized for hub gene(s) selection (63). After that, to find the regulatory genes within the first method, these genes were used to construct the protein-protein interaction (PPI) networks through the Search Tool for the Retrieval Interacting Genes (STRING) database (64) and visualized in Cytoscape v.3.8.2. Only genes with the relationship of high confidence score >0.7 defined as significant were selected to construct the PPI network. Afterward, the nodes (genes) score was calculated using the CytoHubba plug-in Cytoscape (65). Four different centrality methods, including Degree, EcCentricity, Edge Percolated Component (EPC), and Maximum Neighborhood Component (MNC), were used to select the top 10 hub genes for each method. The top 10 hub genes, which fell within these 4 algorithm methods, were chosen as the final set of hub genes (10).

## RESULTS

### Summary of Phenotypic Data

We have previously shown in the Copenhagen sheep model that late gestation and early postnatal malnutrition can induce differential, depot- and sex-specific changes in adipose developmental traits and metabolic outcomes in adulthood, with PER and SUB as the primary targets of prenatal programming in contrast to mesenteric and epicardial adipose tissue (12, 29). By 2.5 yr of age (adulthood), LOW-HCHF sheep had become significantly heavier than LOW-CONV and NORM-CONV, with other groups in between (42). Females, in general, had the highest fat mass (SUB and PER), adipocyte CSA (except for HIGH♀ in PER), CNI (PER only), and proportions of very small (0–40 µm^2^; only PER) as well as medium-large adipocytes (i.e., in SUB: 1,600–3,200 µm^2^ and in PER: 3,200–6,400 µm^2^), but the lowest proportion of small-medium-sized adipocytes (i.e., in SUB: 40–1,600 µm^2^ and in PER: 800–6,400 µm^2^) (29). In PER, the adipocyte density (adipocyte coverage in tissue slides) was highest in females, and HIGH♂ and LOW♂ had the smallest average adipocyte CSA (29). The pattern of distribution of adipocytes on size classes was unimodal in SUB. In contrast, a clear bimodal pattern was observed in PER, with the second peak falling in different adipocyte-size classes depending on treatment groups (29). In PER (not SUB), the numbers of adipocytes categorized as very small (0–40 µm^2^) showed a positive correlation with the numbers of adipocytes in the largest size classes (12,800–36,000 µm^2^) but a negative correlation with numbers in the intermediary adipocyte-size classes (29). In both sexes, LOW-HCHF adult sheep were consistently hypercholesterolemic, hyperuricemic, and hypercreatinemic compared to all other groups (12, 30), and baseline plasma levels (before bolus injections in tolerance tests) of NEFA and TG were also highest in LOW-HCHF sheep.

### Weighted Gene Coexpression Network Analyses

In the present study, we aimed to identify the adipose- and sex-differences gene coexpressed modules that could underline the impacts of different early life nutrition histories (pre- and early postnatal periods) and the associated morphological and metabolic alterations in 2.5-year-old adult sheep. The transcriptomic profiling of the two distinct adipose tissues, SUB and PER, was carried out earlier (29) and was used to create four different gene coexpression networks, namely SUB♂, SUB♀, PER♂, and PER♀ gene networks. The number of modules identified for SUB♂, SUB♀, PER♂, and PER♀ gene networks was 13, 16, 11, and 19, respectively, as shown in Supplemental Fig. S1 (all Supplemental material is available at https://doi.org/10.6084/m9.figshare.20493921).

### Module-Trait Relationships in SUB and PER Adipose Tissues of Adult Sheep as Affected by Pre- and Early Postnatal Nutrition

#### Prenatal nutrition in PER tissue (not SUB) in both sexes showed correlations with coexpression modules associated with cell cycle regulation, gene expression, transmembrane transport, and metabolic processes.

**Figure 1.**
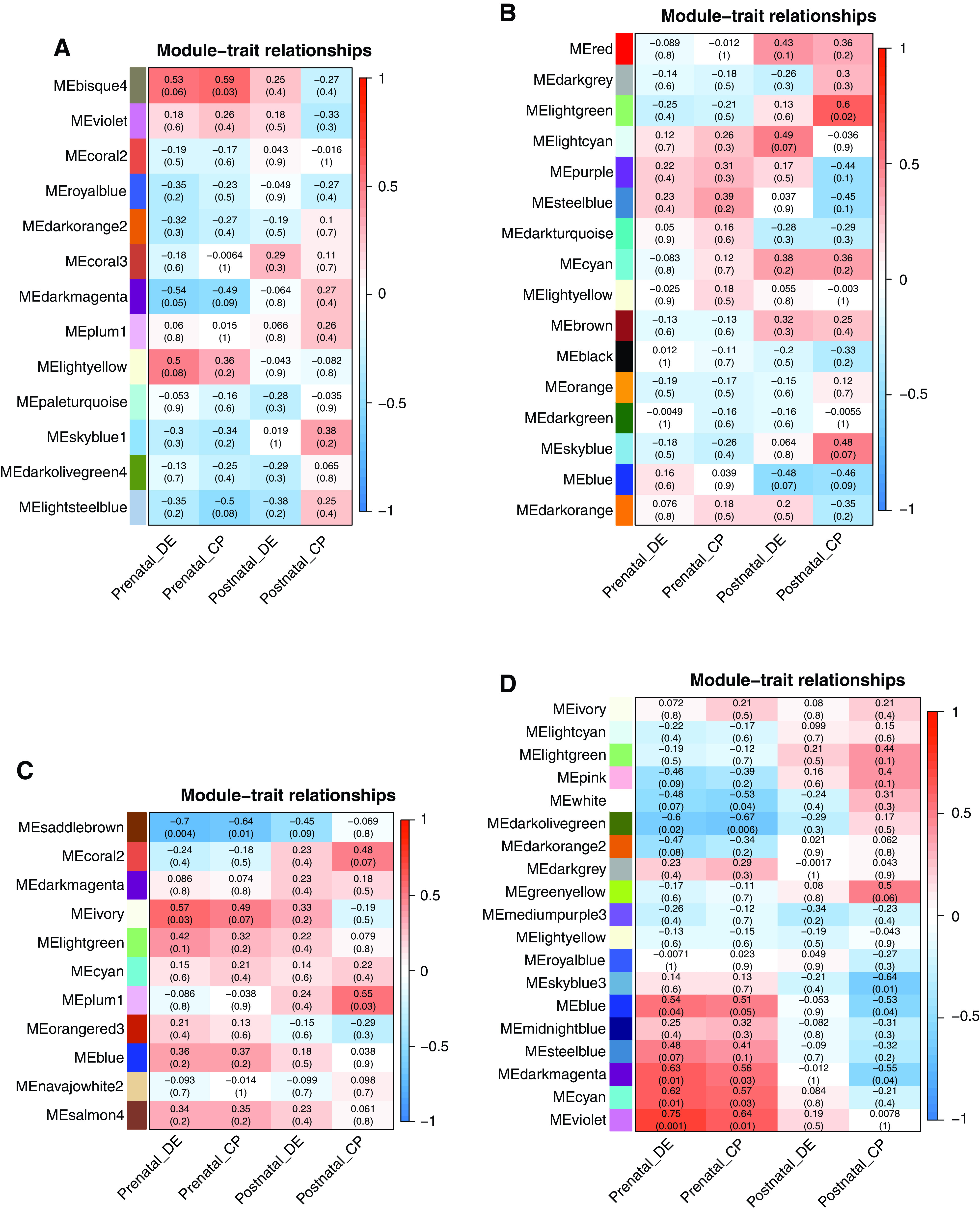
The heat map of module-pre/postnatal supply of digestible energy (DE) and crude protein (CP) relationships for subcutaneous male (SUB♂; *A*; *n* = 13), subcutaneous female (SUB♀; *B*; *n* = 15), perirenal male (PER♂; *C*; *n* = 12), and perirenal female (PER♀; *D*; *n* = 15) networks. Each matrix in the heat map contains a Pearson’s correlation between pre- and early postnatal CP and DE supply (*x*-axis) and module color (*y*-axis) and the corresponding *P* value in parentheses. The module color represents coregulated genes or modules clusters of highly interconnected genes, densely connected subnetworks from gene modules, which are usually related to biological functions. The red and blue colors of the heat map matrix represent positive and negative correlations, respectively, and the more intense the color, the stronger the correlation.

In the constructed PER network, two (“ivory” and “saddlebrown”) and five (“blue,” “cyan,” “darkolivegreen,” “darkmagenta” and “violet”) coexpression modules in PER♂ and PER♀, respectively, were significantly correlated (*P* < 0.05) with prenatal nutrition, i.e., crude protein (CP) or digestible energy (DE) supply to the pregnant dam or both (see Table 1 and Fig. 1 for more details).

**Table 1.** Adipocyte size and correlated modules with their significantly associated functional enrichments from in silico analysis

	Significantly Associated Modules			
	SUB♂	SUB♀	PER♂	PER♀
Adipocyte size class, µm^2^				
0–40	Lightsteelblue ↑ Skyblue1 ↑	Lightcyan ↑	Plum1 **↓**	Midnightblue **↓** Darkolivegreen ↑
40–200		Lightgreen ↑	Saddlebrown ↑	Greenyellow ↑
200–400		Lightgreen ↑	Saddlebrown ↑ Ivory **↓**	
400–800			Saddlebrown ↑ Ivory **↓**	
800–1,600			Saddlebrown ↑	
1,600–3,200			Saddlebrown ↑ Ivory **↓**	Blue ↑
3,200–6,400				Blue ↑
6,400–12,800		Lightgreen**↓**	Saddlebrown **↓** Ivory ↑ Lightgreen **↓**	Lightyellow ↑
12,800–25,600			Saddlebrown **↓** Ivory ↑ Lightgreen **↓**	Blue **↓** Midtnightblue **↓** Darkolivegreen ↑ Steelblue **↓**
25,600–36,000		Lightgreen **↓**	Saddlebrown **↓** Ivory ↑ Plum1 **↓**	Blue **↓** midnightblue **↓** Darkolivegreen ↑ Ivory ↑ Steelblue **↓**
Overview of module enrichments		Tissue development and morphogenesis Microfibril Inflammatory response NAD^+^ binding p38MAPK cascade MicroRNAs Growth and development	Cell division and proliferation/differentiation-related pathways Angiogenesis and vasculogenesis Cell senescence Metabolic pathways Dendritic cell Lipid metabolic process Cell-substrate adhesion Angiogenesis Complement and coagulation Lipid metabolism	DNA integrity checkpoint signaling Ribosome biogenesis Smooth muscle cell ECM remodeling Cytoskeleton Protein synthesis Sterol transport Regulation of fat cell Tumor necrosis factor-mediated signaling DNA/mRNA Cell senescence Metabolic processes

SUB♂, subcutaneous male; SUB♀, subcutaneous female; PER♂, perirenal male; PER♀, perirenal female; ECM, extracellular matrix; ↑, significant positive correlation with the module; ↓, significant negative correlation to the module.

Similar and specific GO terms and pathways in the PER coexpression network were identified that correlated with prenatal nutrition in both sexes. The similar biological processes affected in PER♂ (“saddlebrown”) and PER♀ (“darkolivegreen”) were cell cycle (e.g., cellular response to DNA damage), gene expression, various transmembrane transport systems, and metabolic processes (e.g., of small molecules, macromolecules, and lipids), as shown in Supplemental Tables S3 and S4. However, in PER♀, the enrichments in addition related to, for instance, various transmembrane transport systems (e.g., inorganic molecular entity transmembrane transport, transmembrane transport activity, channel activity, sodium ion transmembrane transporter activity, and amide transmembrane transporter activity) and metabolic processes (e.g., lipid catabolic process, carboxylic acid catabolic process, cellular lipid catabolic process, lipid metabolic process) and were hence more diverse than those seen in PER♂.

Unique to PER♀, mitochondrial organization and functions and extracellular matrix (ECM) remodeling were enriched in all modules significantly associated with prenatal nutrition. The mitochondrion-related processes were particularly identified in the “blue” (mitochondrial inner membrane), “cyan” (mitochondrial matrix organization), “violet” (mitochondrial protein-containing complex), “darkmagenta” module [cellular respiration, mitochondrion, mitochondrial RNA metabolic process, mitochondrial electron transport NADH to ubiquinone, oxidoreductase complex, respiratory chain complex aerobic respiration, oxidoreductase activity acting on NAD(P)H, oxidoreductase activity acting on the CH-CH group of donors]. In addition, the ECM remodeling-related traits were explicitly found in the “blue” (intrinsic component of plasma, intrinsic component of plasma membrane) and “darkolivegreen” (intrinsic component of organelle membrane, integral component of plasma membrane, regulation of extracellular matrix disassembly) modules. Additional enrichments found in the “darkolivegreen” module included Histone H3-K27 trimethylation, Rap1 signaling, skeletal muscle growth, homeostasis of cell numbers, diabetic cardiomyopathy, and type 1 diabetes mellitus.

In contrast, only one module (“bisque4”) enriched in energy metabolism (ADP metabolism) and developmental processes (see Supplemental Table S1) in SUB♂ (not SUB♀) was positively correlated with prepartum CP nutrition of pregnant dams (*r* = 0.59, *P* = 0.03).

### Module-Trait Relationships in SUB and PER Adipose Tissue of Adult Sheep Related to the Adipocyte Size Distribution

#### The proportion of adipocytes in the very small cell-size class (0–40 µm^2^) in the SUB♂/♀ networks and the small perirenal adipocytes (40–200 µm^2^) in the PER♀ network showed positive associations with modules primarily enriched in immunity-related functions.

**Figure 2.**
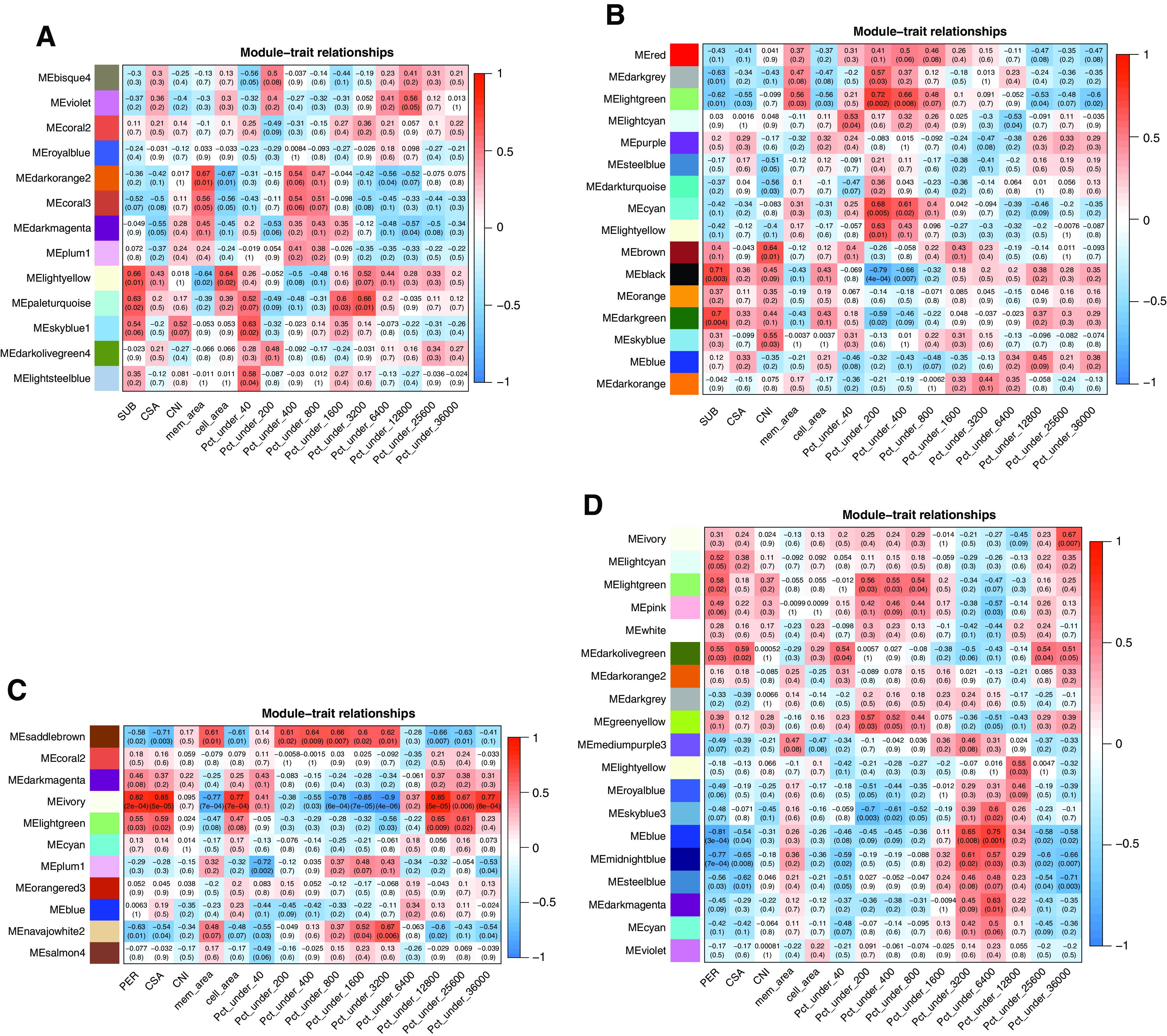
The heat map of module-histomorphometric relationships for subcutaneous male (SUB♂; *A*; *n* = 13), subcutaneous female (SUB♀; *B*; *n* = 15), perirenal male (PER♂; *C*; *n* = 12), and perirenal female (PER♀; *D*; *n* = 15) networks. Each matrix in the heat map contains a Pearson’s correlation and the corresponding *P* value in parentheses between the histomorphometric parameters (*x*-axis) and module color (*y*-axis). The red and blue colors of the heat-map matrix represent positive and negative correlations; the more intense the color, the stronger the correlation. On the *x*-axis of the heat map, SUB and PER represent the weights of subcutaneous and perirenal adipose tissue determined at autopsy. The CSA is the cross-sectional area in histological slides of individual adipocytes, which were automatically categorized by a specially designed application [Iron Hematoxylin Adipose Tissue (APP ID 10113; Visiopharm: protocol 3)] into cell size classes ranging from 0 to 40 (pct_under_40), 40 to 200 (pct_under_200), 200 to 400 (pct_under_400), 400 to 800 (pct_under_800), 800 to 1,600 (pct_under_1600), 1,600 to 3,200 (pct_under_3200), 3,200 to 6,400 (pct_under_6400), 6,400 to 12,800 (pct_under_12800), 12,800 to 25,600 (pct_under_25600), and 25,600 to 36,000 (pct_under_36000) µm^2^. Based on these different cell size classes (0–36,000 µm^2^), two different peaks were groups, namely *peak 1* (0–6,400 µm^2^) and *peak 2* (6,400–36,000 µm^2^). *Protocol 1* of the application calculates the relative percentages of different tissue structures in the slide: adipocytes (cell_area) and membrane area (mem_area). A cell number index was calculated as CNI = (adipose mass (kg) × percentage adipocyte coverage in tissue slides)/volume of a spherical adipocyte. The volume of the spherical adipocyte was calculated using the formula: V = 4/3πr^3^, where the radius, r, was derived from a circle with the same area as the average CSA of adipocytes. The cell size distribution patterns, average CSA, and CNI allowed us to evaluate whether differences in fat deposition resulted from changes in adipocyte numbers (hyperplasia) or size (hypertrophy). Details of the protocols in the Iron Hematoxylin Adipose Tissue (APP ID 10113; Visiopharm) application are described in Ref. 29.

Modules enriched for immunity/cytokine/inflammation-related functions and cellular transport systems in SUB♂, SUB♀, and PER♀ were positively correlated with the proportion of adipocytes in the very small cell-size class (0–40 µm^2^) for SUB♂/♀ and the proportion of small adipocytes (40–200 µm^2^) for PER♀, respectively (see Table 1 and Fig. 2 for more details). These modules were the “lightsteelblue,” “lightcyan,” and “greenyellow” for SUB♂, SUB♀, and PER♀, respectively. In addition, in SUB♂, the “skyblue1” module was positively correlated (*P* < 0.05) with the proportion of very small adipocytes, and this module was significantly enriched in lysosome, phagosome, negative regulation of vascular-associated smooth muscle cell proliferation, lipid storage, osteoclast differentiation, glycolytic process, and glycolipid catabolic process (Supplemental Table S1).

The functional exploration revealed that mitochondrial-related functions constituted a major part of the “lightsteelblue” coexpression module in SUB♂. On the other hand, in PER♀, PPARG signaling and lipid, fatty acid, and cholesterol related-functions (e.g., membrane lipid catabolic process, lipid phosphorylation, high-density lipoprotein particle, lipid metabolic process, fatty acyl-CoA metabolic process, diacylglycerol metabolic process, cholesterol metabolism) were unique for the “greenyellow” module (see more details in Supplemental Table S4).

#### Coexpression modules in SUB♀ and PER ♂/♀ showed associations with the proportion of adipocytes in PER in the large adipocyte size classes (>6, 400 µm^2^) (with sex-specific module enrichments).

One module (“lightgreen”) in SUB♀ and PER♂ networks and three modules (“ivory,” “lightyellow,” and “steelblue”) in PER♀ were significantly correlated (*P* < 0.05) with the proportion of adipocytes in the large cell size classes (>6,400–36,000 µm^2^), as depicted in Fig. 2. The “lightgreen” module in SUB♀ was negatively correlated with the proportion of adipocytes in cell size classes 6,400–12,800 µm^2^ and 25,600–36,000 µm^2^, whereas in PER♂, the “lightgreen” module was negatively correlated with the proportion of adipocytes in cell size classes 6,400–12,800 µm^2^ (*r* = −0.66, *P* = 0.009) and 12,800–25,600 µm^2^ (see Table 1 for more details).

In PER♀, the “ivory” and “lightyellow” modules were positively correlated with the proportions of 25,600–36,000 µm^2^ and 6,400–12,800 µm^2^ sized adipocytes, respectively, whereas the “steelblue” was negatively correlated with the proportions of 12,800–25,600 µm^2^ and 25,600–36,000 µm^2^ sized adipocytes (see Table 1 for more details).

The coexpressed genes in the “lightgreen” module in SUB♀ were enriched for tissue development and morphogenesis (e.g., blood vessel development, circulatory development, embryonic hindlimb morphogenesis, establishment of skin barrier, organ growth, growth factor binding, Wnt-protein binding, regeneration), and others (e.g., microfibril, inflammatory response, NAD+ binding, p38MAPK cascade, MicroRNAs in cancer, etc.) (Supplemental Table S2).

In PER♂, the “lightgreen” module was enriched for cell division and proliferation/differentiation-related pathways (i.e., osteoblast, myoblast, and smooth muscle differentiation, proliferation of smooth muscle), angiogenesis and vasculogenesis (e.g., blood vessel, vasculature development, TGF-beta signaling pathway), cell senescence (e.g., regulation of programmed cell death, muscle cell apoptotic process), and various metabolic pathways that relate to well-documented downstream insulin signaling (e.g., small GTPase-mediated signal transduction, AGE-RAGE signaling pathway in diabetic complication, activation of JUN kinase activity) (Supplemental Table S3).

In PER♀, the “ivory” module was enriched in DNA integrity checkpoint signaling, ribosome biogenesis in eukaryotes, and smooth muscle cell differentiation. The “lightyellow” module was enriched in ECM remodeling (e.g., collagen fibril organization, extracellular matrix, extracellular matrix organization, collagen-binding), cytoskeleton function (e.g., actin filament binding, cilium movement involved in cell motility, actin cytoskeleton reorganization, actin filament), and others (e.g., regulation of smooth muscle migration, renal system development, negative regulation of inflammatory response). The “steelblue” was enriched in protein synthesis (e.g., regulation of protein ubiquitination, regulation of translational initiation, ribosome biogenesis in eukaryotes, ubiquitin-like protein binding), regulation of sterol transport, regulation of fat cell differentiation, tumor necrosis factor-mediated signaling pathway, and DNA/mRNA (e.g., regulation of DNA replication, mRNA surveillance pathway) (Supplemental Table S4).

#### Coexpression modules in PER tissue were correlated with the proportion of adipocytes in the smallest (0–40 µm^2^) as well as largest (25,600–36,000 µm^2^) adipocytes size classes (with sex-specific module enrichments).

In PER, the coexpression network revealed one module in PER♂ (“plum1) and two modules in PER♀ (“midnightblue” and “darkolivegreen”) that were correlated with the proportions of adipocytes in the very small as well as the largest cell-size classes (see Table 1 and Fig. 2 for more details).

According to the functional enrichment analysis, the “plum1” module in PER♂ was enriched in biological processes, such as dendritic cell differentiation, positive regulation of lipid metabolic process, positive regulation of cell-substrate adhesion, cell-cell adhesion via plasma-membrane adhesion molecule, sprouting angiogenesis, and KEGG pathway related to Complement and Coagulation (Supplemental Table S3). In PER♀, the “midnightblue” module showed enrichment in protein-related function, cell senescence processes, RNA/DNA-related processes, and cell proliferation (e.g., regulation of myoblast proliferation, positive regulation of stem cell differentiation) (see more details in Supplemental Table S4).

#### There were opposite patterns of correlation between the adipocyte cell numbers in the peak 1 (0–6,400 µm^2^) and peak 2 (6,400–36,000 µm^2^) cell-size classes with coexpression modules in SUB♀ (not SUB♂) and PER♂/♀.

Few coexpression modules were significantly (*P* < 0.05) associated with the adipocyte numbers in cell size *peak 1* (0–6,400 µm^2^) and cell size *peak 2* (6,400–36,000 µm^2^), and opposite directions of correlations were observed between these two peaks (if *peak 1* showed negative correlation, *peak 2* showed positive correlation and vice versa) as shown in Fig. 2. The involved modules were the “lightgreen” and “blue” in SUB♀ and PER♀, respectively, and in PER♂, the modules were the “saddlebrown” and “ivory” (see Table 1 for more details). The module “lightgreen,” “blue,” and “saddlebrown” for SUB♀, PER♀, and PER♂, respectively, had a positive correlation with the *peak 1* and negative correlations with the *peak 2* adipocyte size classes, whereas the opposite pattern of correlations was seen for the “ivory” module in PER♂ (Fig. 2).

The common significant (*P* < 0.05) trait observed in all these modules was related to ECM remodeling. However, in the PER network (not SUB), the cell cycle was also significantly enriched, specifically in the “saddlebrown” and “blue” modules in PER♂ and PER♀, respectively. Specific to the “lightgreen” module in SUB♀, the unique enrichments were growth and development (e.g., circulatory system development, embryonic hindlimb morphogenesis, morphogenesis of a branching epithelium organ growth, wnt-protein binding), and others (e.g., muscle system process, regeneration, NAD^+^ binding, multicellular organismal homeostasis, p38MAPK cascade, inflammatory response). Specific to the “saddlebrown” module in PER♂ were enrichments in DNA/RNA (e.g., gene expression, RNA processing, RNA polymerase core enzyme, nucleus, nucleic acid binding), mitochondria (e.g., mitochondrion, mitochondrial matrix), lipid (e.g., lipid metabolic process, lipid catabolic process), and others (e.g., regulation of cellular response to stress, fibroblast growth factor binding, MAP kinase). Specific to the “blue” module in PER♀ were various metabolic processes (e.g., nucleic acid metabolic process, purine nucleotide metabolic process, oxoacid metabolic process), protein-related processes (e.g., ribosome, the establishment of protein localization to membrane, spliceosome, protein serine/threonine phosphatase complex, protein localization to microtubule organizing center), lipid metabolism (e.g., lipid catabolic process, lipid homeostasis), the cilium (e.g., cilium movement, 9 + 2 motile cilium), and cell differentiation.

### Module-Trait Relationships in SUB and PER Adipose Tissues of Adult Sheep with Plasma Indicators for Metabolic Adaptability

**Figure 3.**
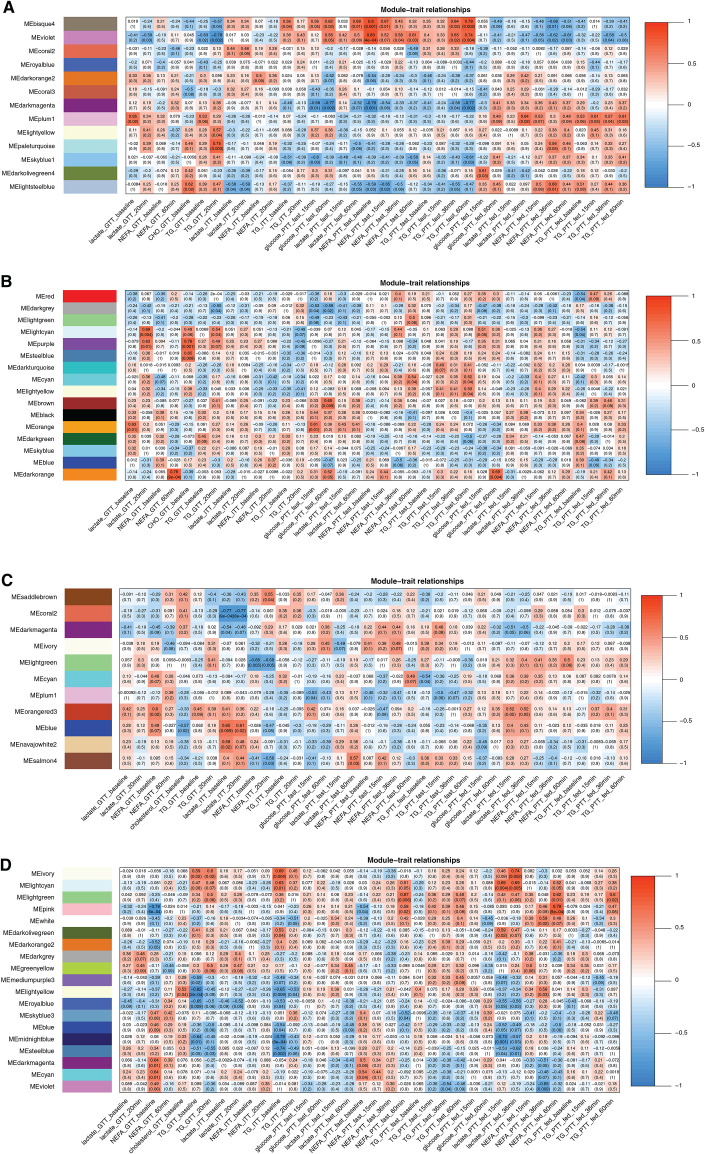
The heat map of module-plasma metabolite concentration relationships in 2.5-yr-old adult sheep for subcutaneous male (SUB♂; *A*; *n* = 13), subcutaneous female (SUB♀; *B*; *n* = 15), perirenal male (PER♂; *C*; *n* = 12), and perirenal female (PER♀; *D*; *n* = 15) networks. The red and blue colors of the heat map matrix represent positive and negative correlations; the more intense the color, the stronger the correlation. Each matrix in the heat map contains a Pearson’s correlation and the corresponding *P* value in parentheses between module color (*y*-axis). Concentrations of plasma metabolites (*x*-axis) measured in tolerance tests at different time points (in minutes) before (basal) or after intravenous bolus injections of either glucose (GTT), insulin (ITT), or propionate, the latter conducted in both fed (PTT_fed) and fasted (PTT-fasted) states. The determined plasma metabolites were glucose, nonesterified fatty acids (NEFA), triglycerides (TG), blood urea nitrogen, creatinine, lactate, β-hydroxybutyrate (BOHB), γ-glutamyl transferase (GGT), and cholesterol.

All correlations between modules and plasma concentrations of metabolites and hormones measured in intravenous tolerance tests are available in Table 2. We only observed correlations between coexpressed modules and plasma levels of cholesterol and basal levels (before any intravenous bolus injections in tolerance tests) of NEFA and TG (Fig. 3). For plasma cholesterol, plasma levels measured in the GTT correlated to one coexpression module in both SUB♂ (“lightsteelblue), PER♂ (“blue”), and PER♀ (“lightyellow”) and to two modules in SUB♀ (“purple” and “steelblue”) (see Table 2 for more details).

**Table 2.** Plasma metabolites and correlated modules with their significantly associated functional enrichments

	Significantly Associated Modules			
Plasma Metabolites	SUB♂	SUB♀	PER♂	PER♀
Basal plasma TG	Plum ↑	Lightcyan **↓**	Cyan **↓**	Blue **↓** Lightyellow **↓** Steelblue **↓** Royalblue **↓**
Basal plasma cholesterol	Lightsteelblue ↑	Purple ↑ Steelblue ↑	Blue **↓**	Lightyellow ↑
Basal NEFA level				Cyan ↑ Darkmagenta ↑
T_4_ level in iTTT (8 and 15 min postinjection)				Cyan ↑ Darkmagenta ↑ Pink **↓**
TSH level in iTTT at 8 and 15 min postinjection)				Ivory ↑
Overview of module enrichments		Metabolic signaling pathways (e.g., positive regulation of MAPK cascade, regulation of NF-κB transcription) Gonadotropin releasing hormone (GnRH) secretion	Metabolic processes, ECM, mitochondria-related functions, cellular response to DNA damage, and DNA damage checkpoint Negative regulation of B-cell proliferation, cellular response to lipoprotein particles, and cellular response to lipoprotein particles (cyan)	Blood vessel development and cholesterol metabolism Regulation of sterol transport, regulation of fat cell differentiation, and others Mitochondria-related functions chain complex Epigenetics (i.e., chromatin organization, histone H3-K4 trimethylation, and histone deubiquitination DNA integrity checkpoint signaling, ribosome biogenesis in eukaryotes, and smooth muscle cell differentiation

The concentrations of plasma metabolites were measured in tolerance tests at different time points (in minutes) before (basal) or after intravenous bolus injections of either glucose, insulin, or propionate, the latter conducted in both fed and fasted states. The determined plasma metabolites were glucose, nonesterified fatty acids (NEFA), triglycerides (TG), blood urea nitrogen, creatinine, lactate, β-hydroxybutyrate, γ-glutamyl transferase, and cholesterol. SUB♂, subcutaneous male; SUB♀, subcutaneous female; PER♂, perirenal male; PER♀, perirenal female; ECM, extracellular matrix; iTTT, intravenous thyroxine tolerance test; TSH, thyroid-stimulating hormone; T_4_, thyroxine; ↑, significant positive correlation with the module; ↓, significant negative correlation with the module.

Basal plasma levels of TG correlated with coexpression module(s) in both adipose tissues and sexes, namely in SUB♂ (“plum1” in PTT_fed), SUB♀ (“lightcyan” in PTT_fed), PER♂ (“cyan” in PTT_fast), and PER♀ (“blue,” “lightyellow,” “royalblue,” and “steelblue” in ITT) (see Table 2 for more details).

For PER♀ specifically, two coexpression modules were associated with basal plasma levels of NEFA (in GTT for both “cyan” and “darkmagenta”).

For the “cyan” module in PER♂ as well as the three modules “lightcyan,” “purple,” and “steelblue” in SUB♀ (*P* < 0.05), the primary biological terms present were related to immune and inflammatory functions (see more details in Supplemental Tables S3 and S4). In SUB♀, the enriched pathways in the “purple” module were related to metabolic signaling pathways (e.g., positive regulation of MAPK cascade, regulation of NF-κB transcription) as well as gonadotropin releasing hormone (GnRH) secretion. For PER♂, enrichments found in the “cyan” module were related to negative regulation of B cell proliferation, cellular response to lipoprotein particles, and cellular response to lipoprotein particles.

The “blue” module in PER♂ was enriched for various metabolic processes, ECM, mitochondria-related functions, cellular response to DNA damage, and DNA damage checkpoint (see more details in Supplemental Table S3). Specific to PER♀, some of the important functional enrichments in the “royalblue” module were blood vessel development and cholesterol metabolism, and the “steelblue” module included regulation of sterol transport, regulation of fat cell differentiation, and others (e.g., regulation of protein ubiquitination, ribosome biogenesis in eukaryotes, tumor necrosis factor-mediated signaling pathway, regulation of DNA replication and mRNA surveillance pathway).

**Figure 4.**
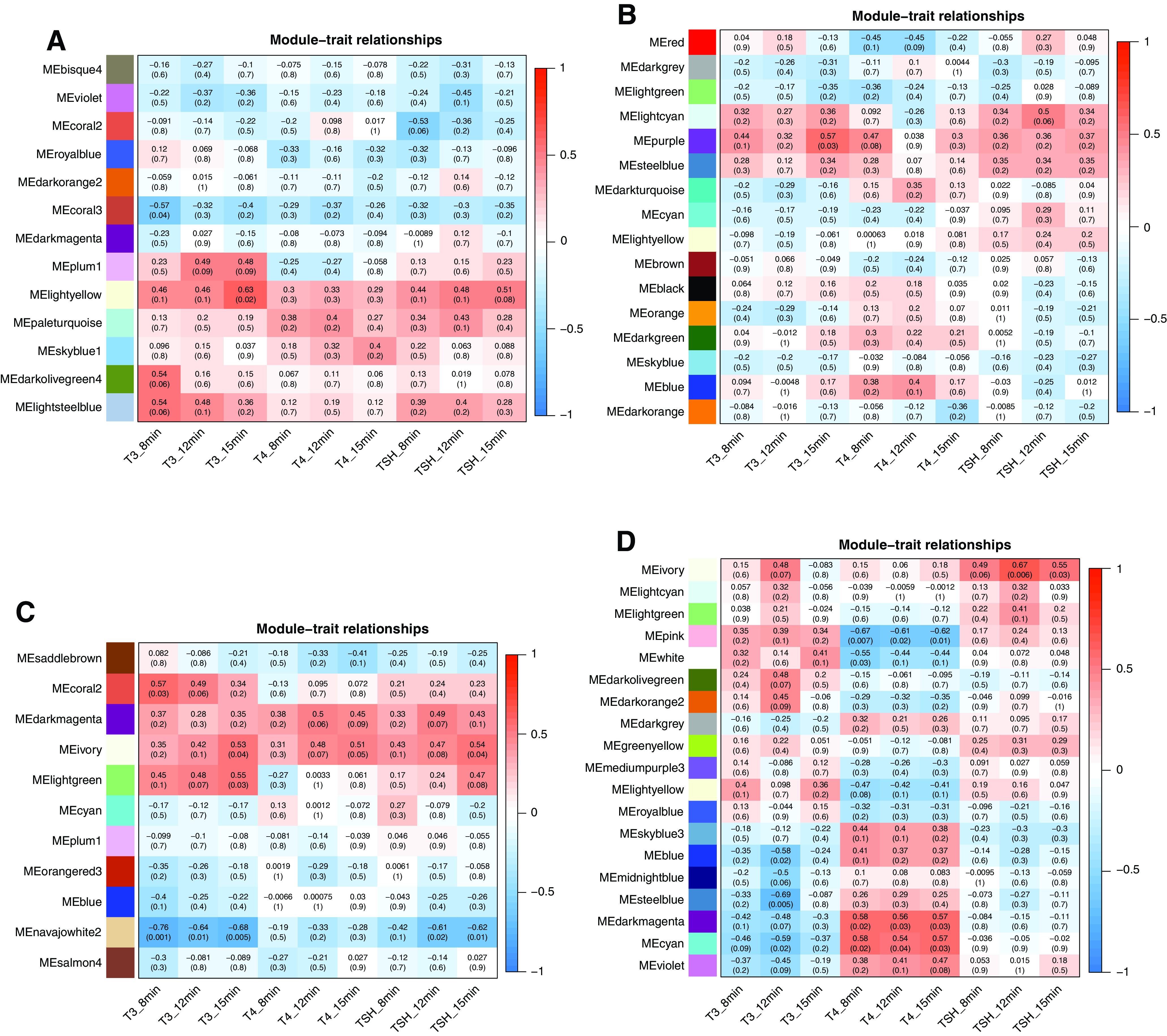
The heat map of module-thyroid hormone axis function-related relationships for subcutaneous male (SUB♂; *A*; *n* = 13), subcutaneous female (SUB♀; *B*; *n* = 15), perirenal male (PER♂; *C*; *n* = 12), and perirenal female (PER♀; *D*; *n* = 15) networks. Each matrix in the heat map contains a Pearson’s correlation and the corresponding *P* value in parentheses between module color (y-axis) and serum concentrations of thyroid hormones (T_4_, thyroxine; T_3_, triiodothyronine) and thyroid stimulating hormone (TSH) (*x*-axis). The red and blue colors of the heat map matrix represent positive and negative correlations, respectively; the more intense the color, the stronger the correlation. The serum levels of T_3_, T_4_, and TSH were determined in 2.5-yr-old adult sheep at different time points during 2 days in response to an intravenous injection of T_4_ in a thyroxine tolerance test (iTTT) following a period of overnight fasting.

In the PER♀ coexpression network, three modules, namely the “cyan,” “darkmagenta,” and “pink,” were correlated to serum levels of T_4_ in the iTTT determined at different time points after the bolus injection of T_4_. Positive correlations were found for “cyan” and “darkmagenta” and negative correlations for the “pink” module (see Table 2 and Fig. 4 for more details). Both the “cyan” and “darkmagenta” modules were enriched in mitochondria-related functions (“cyan”: xelectron transport chain and mitochondrial matrix; “darkmagenta”: mitochondrial RNA metabolic process, mitochondrial electron transport, NADH to ubiquinone, oxidoreductase complex and respiratory chain complex). In contrast, epigenetics such as chromatin organization, histone H3-K4 trimethylation, and histone deubiquitination were enriched in the “pink” module.

Finally, the “ivory” module in the PER♀ coexpression network, enriched for DNA integrity checkpoint signaling, Ribosome biogenesis in eukaryotes, and smooth muscle cell differentiation, was positively correlated with serum levels of TSH measured in the iTTT (see Table 2 for more details).

### Hub Genes Identification via Protein-Protein Interaction Networks and Intramodular Connectivity (Module Membership)

Several hub genes were identified from modules that correlated with prenatal nutrition (CP and DE supply to the mother during pregnancy), as shown in Table 3. The *SPAG5* is a hub gene specifically in the “plum1”, “purple,” and “blue” module in the coexpression networks of SUB♂, SUB♀, and PER♂. In addition, the *AURKA* gene was identified as a hub gene in the modules “blue” in PER♂ and “darkolivegreen” in PER♀. *ITGB2*, *CTSS*, and *TYROBP* were hub genes in the “cyan” module in the PER♂ coexpression network.

**Table 3.** Module-trait relationships and identified hub genes in modules that correlated with phenotypic traits for each network, namely SUB♂, SUB♀, PER♂, and PER♀

Module	Early Nutrition, Plasma Metabolite, or Adipose Cell Size Class	Module-Trait Relationships	Hub Genes	Protein Name
SUB♂				
Bisque4	Prenatal CP	*r* = 0.59, *P* = 0.03		
Lightsteelblue	0–40 µm^2^ Basal plasma cholesterol level	*r* = 0.58, *P* = 0.04 *r* = 0.62, *P* = 0.02		
Lightgreen	6,400–12,800 µm^2^ 25,600–36,000 µm^2^	*r* = −0.53, *P* = 0.04 *r* = −0.60, *P* = 0.02		
Plum1	Basal plasma TG level	*r* = 0.61, *P* = 0.03	*HMMR*, *TTK*, *CENPF*, *DLGAP5*, *MKI67*, *ASPM*, *CENPE*, *KIF20A*, *SPAG5*	Hyaluronan-mediated motility receptor (RHAMM), protein kinase domain-containing protein, centromere protein F, DLG associated protein 5, FHA domain-containing protein, abnormal spindle-like microcephaly-associated protein homolog, centromere protein E, kinesin-like protein, sperm associated antigen 5
skyblue1			*LCP2*	SH2 domain-containing protein
SUB♀				
Lightcyan	0–40 µm^2^ Basal plasma TG level	*r* = 0.53, *P* = 0.04 *r* = *−*0.54, *P* = 0.04	*CCNB2*	Cyclin NH_2_-terminal domain-containing protein
Skyblue1	0–40 µm^2^	*r* = 0.63, *P* = 0.02		
Lightgreen	6,400–12,800 µm^2^ 25,600–36,000 µm^2^ Cell size *peak 1* (0–6,400 µm^2^) Cell size *peak 2* (6,400–36,000 µm^2^)	*r* = −0.53, *P* = 0.04 *r* = −0.60, *P* = 0.02 40–200 µm^2^: *r* = 0.72, *P* = 0.002 200–400 µm^2^: *r* = 0.66, *P* = 0.008 25,600–36,000 µm^2^: *r* = *−*0.60, *P* = 0.02	*ACAA1*, *MRPL24*	Acetyl-CoA acyltransferase 1, KOW domain-containing protein
Purple	Basal plasma cholesterol level	*r* = 0.76, *P* = 0.001	*HJURP*, *PLK1*, *KIFC1*, *KIFC2*, *CENPN*, *SPAG5*, *E2F8*	Holliday junction recognition protein, serine/threonine-protein kinase PLK, kinesin-like protein 1, kinesin-like protein C2, centromere protein N, sperm associated antigen 5, E2F transcription factor 8
Steelblue	Basal plasma cholesterol level	*r* = 0.65, *P* = 0.008		
PER♂				
Ivory	Prenatal CP Cell size *peak 2* (6,400–36,000 µm^2^)	*r* = 0.57, *P* = 0.03 200–400 µm^2^: −0.54, *P* = 0.03 400–800 µm^2^: −0.78, *P* = 0.0006 1,600–3,200 µm^2^: *r* = −0.90, *P* = 4.04^e-06^ 6,400–12,800 µm^2^: *r* = 0.85, *P* = 5.07^e-05^ 12,800–25,600 µm^2^: *r* = 0.67, *P* = 0.006 25,600–36,000 µm^2^: *r* = 0.77, *P* = 0.0008		
Saddlebrown	Prenatal DE Prenatal CP Cell size *peak 1* (0–6,400 µm^2^) Cell size *peak 2* (6,400–36,000 µm^2^)	*r* = *−*0.7, *P* = 0.004 *r* = *−*0.64, *P* = 0.01 40–200 µm^2^: *r* = 0.61, *P* = 0.02; 200–400 µm^2^: *r* = 0.64, *P* = 0.009; 400–800 µm^2^: *r* = 0.66, *P* = 0.007; 800–1,600 µm^2^: *r* = 0.61, *P* = 0.02; 1,600–3,200 µm^2^: *r* = 0.62, *P* = 0.01 6,400–12,800 µm^2^: *r* = −0.66; *P* = 0.007 12,800–36,000 µm^2^: *r* = −0.63, *P* = 0.01		
Lightgreen	>6,400 µm^2^	6,400–12,800 µm^2^: *r* = −0.66, *P* = 0.009 12,800–25,600 µm^2^: *r* = −0.63, *P* = 0.02	*NOL6*, *RSL1D1*, *GRWD1*, *POLR1A*, *PUM3*, *DDX10*, *RRP12*, *GNL3L*, 9940.ENSOARP00000009272	Nucleolar protein 6, ribosomal L1 domain containing 1, WD_REPEATS_REGION domain-containing protein, DNA-directed RNA polymerase subunit, PUM-HD domain-containing protein, RNA helicase, uncharacterized protein, mRNA turnover 4 homolog (*S. cerevisiae*)
Lightyellow	Basal plasma TG level	*r* = *−*0.54, *P* = 0.04		
Lightgreen				
Plum1	0–40 µm^2^ 25,600–36,000 µm^2^	*r* = −0.72, *P* = 0.002 *r* = −0.53, *P* = 0.04	*MAOB*, 9940.ENSOARP00000000261, *ALDH2*, *TIMELESS*, *WWF*, *SLC26A2*	Amine oxidase, amine oxidase, Aldedh domain-containing protein, Ovis aries timeless (TIM), von Willebrand factor, Sulfate transporter (Solute carrier family 26 member 2)
Blue	Basal plasma cholesterol level	*r* = −0.57, *P* = 0.02	*RRM2*, *AURKA*, *SPAG5*	RRM2 - ribonucleotide reductase regulatory subunit M2
Cyan	Basal plasma TG level	*r* = −0.54, *P* = 0.04	*SPI1*, *ITGB2*, *CTSS*, *TYROBP*	SPI1 - Spi-1 proto-oncogene b
PER♀				
Blue	Prenatal DE Cell size *peak 1* (0–6,400 µm^2^) Cell size *peak 2* (6,400–36,000 µm^2^) Basal plasma TG level	*r* = 0.54, *P* = 0.04 1,600–3,200 µm^2^: *r* = 0.65, *P* = 0.006 3,200–6,400 µm^2^: 0.75, *P* = 0.001 12,800–25,600 µm^2^: −0.58, *P* = 0.02 25,600–36,000 µm^2^: *r* = −0.58, *P* = 0.02 *r* = *−*0.54, *P* = 0.04		
Cyan	Prenatal DE Prenatal CP Basal plasma NEFA level T_4_ in the iTTT	*r* = 0.62, *P* = 0.01 *r* = 0.57, *P* = 0.03 *r* = 0.64, *P* = 0.01 at 8–15 min postinjection: *r* = −0.4– 0.58, *P* = 0.02 – 0.04	*SDHC*, *COX5A*, *CYC1*	Succinate dehydrogenase cytochrome b560 subunit, mitochondrial, cytochrome *c* oxidase polypeptide Va (cytochrome *c* oxidase subunit 5 A, mitochondrial), cytochrome c domain-containing protein
Darkolivegreen	Prenatal DE Prenatal CP Very small-cell size (0–40 µm^2^) Largest cell-size (12,800–36,000 µm^2^)	*r* = −0.6, *P* = 0.02 *r* = −0.67, *P* = 0.006 0–40 µm^2^: *r* = 0.54, *P* = 0.03 12,800–25,600 µm^2^: *r* = 0.54, *P* = 0.04 25,600–36,000 µm^2^: *r* = 0.51, *P* = 0.049	*AURKA*	Aurora kinase
Darkmagenta	Prenatal DE Prenatal CP Basal plasma NEFA level T_4_ in the iTTT	*r* = 0.63, *P* = 0.01 *r* = 0.56, *P* = 0.03 *r* = 0.64, *P* = 0.01 at 8–15 min postinjection: *r* = −0.6– 0.58, *P* = 0.02 – 0.03		
Violet	Prenatal DE Prenatal CP	*r* = 0.75, *P* = 0.001 *r* = 0.64, *P* = 0.01	*NDUGAB1*, *PSMB1*	Acyl carrier protein, proteasome subunit beta
Greenyellow			*PTPRC*	Protein-tyrosine-phosphatase
Ivory	25,600–36,000 µm^2^	*r* = 0.67, *P* = 0.007	*MPHOSPH10*, *MZB1*, 9940.ENSOARP00000013486, *ATL1*, *POLD4*, *FDX2*, *LIMD2*, *NGDN*, *JCHAIN*, *VRK3*	U3 small nucleolar ribonucleoprotein protein MPP10, DUF3456 domain-containing protein, uncharacterized protein, GB1/RHD3-type G domain-containing protein, Uncharacterized protein, 2Fe-2S ferredoxin-type domain-containing protein, LIM zinc-binding domain-containing protein, neuroguidin, EIF4E binding protein, joining chain of multimeric IgA and IgM; immunoglobulin J polypeptide, linker protein for immunoglobulin alpha and mu polypeptides, protein kinase domain-containing protein
Lightyellow	6,400–12,800 µm^2^ Basal plasma TG level	*r* = 0.55, *P* = 0.03 *r* = −0.65, *P* = 0.009	*ACTA2*, *MYH11*, *ACTG2*, *TPM1*, *TPM4*, *TPM2*, *ACTN1*, *TAGLN*, *MYL9*	Uncharacterized protein, myosin, heavy chain 11, smooth muscle, ACTG2, Ovis aries tropomyosin 1 (alpha), tropomyosin 4, tropomyosin 2 (beta), actinin, alpha 1, transgelin, myosin light chain 9
Steelblue	12,800–25,600 µm^2^ 25,600–36,000 µm^2^ Basal plasma TG level	*r* = −0.54, *P* = 0.04 *r* = −0.71, *P* = 0.003 *r* = −0.74, *P* = 0.002	*NOP58*, *NIFK*, *DDX5*, *GTPBP4*, *UTP6*, *DNTTIP2*, *WDR75*	Nop domain-containing protein, RRM domain-containing protein, DEAD box protein 5, nucleolar GTP-binding protein 1, uncharacterized protein, Fcf2 domain-containing protein, WD repeat domain 75
Midnightblue	0–40 µm^2^ 12,800–25,600 µm^2^ 25,600–36,000 µm^2^	*r* = −0.59, *P* = 0.02 *r* = −0.60, *P* = 0.007 *r* = −0.66, *P* = 0.04	*SETD2*	SET domain-containing protein 2
Saddlebrown				
Royalblue	Basal plasma TG level	*r* = −0.66, *P* = 0.009	*FMR1*, *BAK1*, *LYPLA2*	Synaptic functional regulator FMR1, BCL2-antagonist/killer 1, abhydrolase_2 domain-containing protein
Pink	T_4_ in the iTTT	at 8–15 min postinjection: −0.67–0.61, *P* = 0.02–0.07		

Prenatal DE and prenatal CP, prepartum supply of digestible energy and crude protein to the pregnant dam. The proportion of adipocytes categorized in cell size classes ranging from 0 to 40 (pct_under_40), 40 to 200 (pct_under_200), 200 to 400 (pct_under_400), 400 to 800 (pct_under_800), 800 to 1,600 (pct_under_1600), 1,600 to 3,200 (pct_under_3200), 3,200 to 6,400 (pct_under_6400), 6,400 to 12,800 (pct_under_12800), 12,800 to 25,600 (pct_under_25600), and 25,600 to 36,000 (pct_under_36000) µm^2^, respectively (see more details in Fig. 3 legend). Based on these different cell size classes (0–36,000 µm^2^), 2 different peaks were groups, namely *peak 1* (–6,400 µm^2^) and *peak 2* (6,400–36,000 µm^2^). Plasma levels of nonesterified fatty acids (NEFA), triglycerides (TG), and cholesterol levels were measured during so-called intravenous tolerance tests, i.e., before and after intravenous bolus injections of glucose, insulin, and propionate (the latter test conducted both during the fed state: PTT_fed; and fasted state: PTT_fast) (see more details in Fig. 3 legend). Serum level of thyroxine (T_4_) was determined during an intravenous thyroxine tolerance test (iTTT) following overnight fasting and conducted over 2 days (see more details in Fig. 4 legend). Hub genes were selected based on the module membership (MM) and using the CytoHubba application. Genes with the highest MM (MM ≥0.8 and *P* < 0.05) in each selected module significantly correlated to at least 1 trait of interest were prioritized for hub genes selection. After that, the protein-protein interaction networks were constructed using these genes (only genes with the relationship of high confidence score >0.7), followed by nodes (genes) score calculation using the CytoHubba through 4 centrality methods (degree, EcCentricity, edge percolated component, and maximum neighborhood component). The top 10 genes within these 4 centrality methods were selected as hub genes. SUB♂, subcutaneous male; SUB♀, subcutaneous female; PER♂, perirenal male; PER♀, perirenal female.

## DISCUSSION

This study has provided insight into the transcriptional and biological mechanisms underlying the long-term implications of different pre- and early postnatal malnutrition exposures, which can contribute to explaining changes in adipose expandability traits and any associated predisposition for metabolic disturbances later in life. We created four independent gene coexpression networks using our previously published gene expression datasets from SUB and PER of adult sheep from the Copenhagen sheep model (10). We then correlated them to previously studied parameters (phenotypic traits) in the same sheep. The *P* values of the MTR were plots to check if the Pearson for function in WGCNA is prone to outliers. We observed that the distributions of *P* values were as expected for the PER female network, whereas the *P* value distributions for other gene networks (PER male, SUB female, and SUB male) were uniform. These *P* values were subsequently corrected using the FDR approach, and the modules with an FDR value *P* < 0.05 were considered significant.

Long-term effects of prenatal nutrition (prepartum DE and CP supply) on PER had implications for fundamental developmental and functional biological processes through long-term programming of molecular mechanisms, such as cell cycle regulation, gene expression, transmembrane transport, and metabolism in both sexes (♂/♀). In contrast, only an association to adenosine diphosphate metabolic process and development/morphogenesis (e.g., mammary gland and tube closure) was observed in SUB♂. Additionally, we demonstrated sex-specific implications of prenatal nutrition in PER, where chromatin was specific to PER♂, whereas mitochondria- and OXPHOS-related functions and H3-K27 trimethylation were specific to PER♀. The present study’s findings extend previous observations that there is evidence of sexually dimorphic responses to early life programming (66, 67).

We have previously documented for PER specifically that there was a significant positive correlation between the proportions of adipocytes characterized as very small (0–40 µm^2^) to the proportion of very large adipocytes (25,600–36,0000 µm^2^), whereas the correlations between very small adipocytes to the proportions of adipocytes in intermediary cell-size classes (800–12,800 µm^2^) were negative (29). In addition, there was a shift in the distribution of adipocytes across cell size classes in HIGH♂ compared to LOW/NORM♂, and the LOW♀ was found to have the highest adipocyte expandability compared to NORM/HIGH♀ and all males (29). There are conflicting observations about whether adipocyte size (small vs. large) and anatomical location (fat depots) can predict metabolic dysfunction risks. Experimental data from obese humans (68) and canine animal models of diet-induced insulin resistance (69) have demonstrated that the propensity for adipocyte expandability by hypertrophy is associated with an increased risk of insulin resistance. However, there are still inconsistent reports regarding which type(s) of the fat depot(s) contribute(s) in particular to this insulin resistance. In obese humans, large adipocytes in SUB had a closer association with lower plasma insulin levels than larger visceral adipocytes (68). In contrast, in the canine model, the appearance of hypertrophic adipocytes within visceral adipose tissue (>75 µm) was a more critical predictor of insulin resistance than larger SUB adipocytes (69).

Other studies have demonstrated that small adipocytes are often considered healthier than large hypertrophied adipocytes since secretions of various cytokines are often increased when adipocytes grow large as fat accumulates (20). Nevertheless, several other studies have shown that both the occurrence of a special subpopulation of very small adipocytes (<40 µm) in SUB and omental fat, as well as the presence of large hypertrophied adipocytes in SUB, is correlated to the degree of insulin resistance in obese humans (18, 70, 71) and type II diabetes in Asian Indians (2). A previous study in a sheep model of malnutrition has also demonstrated a greater preponderance of very small adipocytes (<1,250 µm^2^ equivalent to <40 µm) in the SUB of LOW sheep (72) with reduced insulin sensitivity (73). Once adipocytes reach a certain “threshold” or capacity for fat storage, hypertrophy of the population of smaller adipocytes is normally expected to occur, but the subpopulation of very small adipocytes appears to have an impaired ability to proliferate and mature into fully functional adipocytes and/or failure in their ability to accumulate lipids (18, 71). Through WGCNA, we found a significant positive association between the population of very small (0–40 µm^2^) and very large (25,600–36,000 µm^2^) adipocyte size classes in PER♀ with coexpressed genes in the “darkolivegreen” module, which were associated with diabetic cardiomyopathy, type 1 diabetes mellitus, and RAP1 signaling, whereas another module, “lightgreen,” enriched for AGE-RAGE signaling pathway in diabetic complication was significantly positively correlated with only very large adipocytes (12,800–25,600 µm^2^) in the PER♂ gene network. The eigengene expression was upregulated across samples within the prenatal LOW♀ and HIGH♂ in the “darkolivegreen” and “lightgreen” modules, respectively, as shown in Supplemental Fig. S2. Taken together, previous findings (2, 18, 68–71) and our study support the notion that a population of very small adipocytes and especially extensive hypertrophy of other adipocytes might be associated with diabetic complications. Thus female and male sheep were found to be susceptible to long-term implications of prenatal LOW and HIGH nutrition predisposing for a risk fact by increasing PER adipocyte hypertrophic ability.

The mitochondria generate cellular energy required for the metabolism of cells and tissues, primarily in the form of ATP via the flow of protons in the electron transport chain (74). The mitochondria also serve as a signaling system, where they participate in the regulation of cell fate, differentiation, and apoptosis, control of canonical developmental signaling pathways (e.g., Notch, NF-κb, and Wnt signaling), and serve as a site of essential metabolism (e.g., amino acid, steroid, cholesterol, and phospholipid biosynthesis) and other functions (e.g., control of intracellular Ca^2+^, cellular redox potential, and reactive oxygen species production) (74). Based on substantial evidence from animal and human studies, Gyllenhammer et al. (74) proposed that the magnitude and consequences of exposures that affect the functional development of mitochondria are more pronounced during intrauterine life since alterations in cellular biology occurring early in life are likely to have greater accumulated consequences than those occurring later in life. In line with this, numerous studies have demonstrated that mitochondrial changes induced by adverse exposures during gestation persist into adulthood (75, 76) and may even be heritable across generations (77). Although these previous studies have focused mainly on mitochondrial functions in skeletal muscle, our results suggest that late gestation malnutrition is also associated with impaired mitochondrial-related functions in adipose tissue, particularly in PER and females.

We have previously shown that LOW♀ had higher numbers of very large adipocytes in PER than NORM + HIGH♀ (29). Using WGCNA, specific for PER♀, mitochondria- and OXHPOS-related functions were found to be enriched (correlated to prenatal nutrition), and there was an association between proportions of very small/large adipocytes with disease-related modules (enriched in the homeostasis of cell numbers, thyroid hormone signaling, type 1 diabetic mellitus, and diabetic cardiomyopathy). Obese adipose tissues have been shown to exhibit downregulation of genes related to mitochondrial metabolism and functions, including OXPHOS, fatty acid beta-oxidation, and mitochondrial oxidative capacity (78). Congruently, in the present study, the concomitant appearance of very small and large adipocytes, and a general shift in PER adipocyte cell size distribution toward more very large PER adipocytes in LOW♀ particularly, could be associated with impaired mitochondria and OXPHOS-related functions. This supports that prenatal programming of PER resulting in reduced hyperplasic ability could be associated with an increased risk for type II diabetes later in life (denoted by overexpression of genes related to this disease in LOW♀).

In contrast to LOW♀ and HIGH♂, who had increased PER adipocyte hypertrophic ability, LOW♂ had the lowest hypertrophic adipocyte expandability (29). Specifically, there was a shift within cell size *peak 2* in PER from the populations of largest (6,400–25,600 µm^2^) to medium-sized (800–3200 µm^2^) adipocyte classes in LOW♂ (29). To find the molecular mechanisms contributing to this shift in PER adipocyte size in LOW♂, we looked for the module(s) that showed significant association with the adipocyte size classes of 800–3,200 µm^2^ and 6,400–25,600 µm^2^. In the PER♂ gene network, the “saddlebrown” module enriched for chromatin modification, gene expression, and cell cycle showed a significant positive association with the proportion of medium-sized (800–3,200 µm^2^) adipocytes and negatively correlated to the proportion of large (6,400–25,600 µm^2^) adipocyte size classes. The eigengene expression of this module showed overexpression in LOW♂ compared to NORM/HIGH♂ samples. Fat mass increase involves preadipocyte (or immature adipocyte) proliferation and adipogenic differentiation, with the former requiring cell cycle activity and the latter involving cell cycle inhibition (79). The adipocyte differentiation is controlled by chromatin-modifying enzymes, such as histone acetyl-transferases (HAT1 and KAT2B) and histone deacetylase (HDAC1), that act as transcriptional activators and suppressors during cellular differentiation, respectively (80, 81). For instance, the knockdown of *HDCA1* promotes adipogenesis, whereas *HDAC1* overexpression attenuates adipocyte differentiation in 3T3-L1 cells (82). Likewise, another study has shown that *HDC1* expression decreases along with the differentiation process, and *HADC1* is known to inhibit *PPARG* and its target genes involved in adipocyte differentiation (83). Moreover, increased overexpression of cell cycle inhibitors, such as cyclin-dependent kinase inhibitor 2 C, *CDKN2C*, and peripheral myelin protein 22 (*PMP22*), is also required for adipocyte differentiation (79).

As mentioned previously, we demonstrated that the sex-specific module correlations to prenatal nutrition specific to PER♂ were enriched for chromatin (containing *HDAC1*) and cell cycle-related processes, such as the G_1_/S transition of the mitotic cycle (*CDKN1A*, *CDKN1B*, *CDKN2C*, *E2F3*, and *E2F4* among the involved genes) and nuclear cyclin-dependent protein kinase. In addition, the expression profile of genes involved in these biological pathways showed a clear overexpression in LOW♂ but underexpression in HIGH♂. In line with previous studies (82, 83), overexpression of the adipocyte differentiation activator *HDAC1* might be an underlying molecular change or mechanism responsible for the reduced hypertrophic expandability (impaired adipocyte differentiation and maturation) observed in LOW♂ and increased hypertrophic expandability in HIGH♂ (denoted by underexpression of *HDAC1*). Although overexpression of cell cycle inhibitors, such as *CDKN2C* required for adipocyte differentiation, was also observed in LOW♂, it was apparently insufficient to result in hypertrophic growth of PER adipocytes in LOW♂.

It has previously been shown that human mesenchymal stem cells express various inflammatory cytokines such as interleukin-beta 1 (*IL1B*), interleukin-6 (*IL6*), tumor necrosis factor-alpha (*TNFA*), and interferon-gamma (*IFNγ*), which can block their differentiation into mature adipocytes (84). Likewise, three-dimensional culture studies with human adipose tissue-derived stem cells indicate that expression of *TNFA*, *IFNγ*, and interleukin-10 (*IL10*) is high during the early stages of adipogenesis (81). Still, the secretion of these cytokines decreases during the differentiation process with the progression of preadipocytes into adipocyte-like cells (85). The present study also found that immunity/cytokines/inflammation modules were positively correlated with the proportion of adipocytes in the size class of very small adipocytes (0–40 µm^2^) in all gene networks except PER♂.

At the age of 2.5 yr, the LOW-HCHF sheep (of both sexes) became hypercholesterolemic, hyperuremic, and hypercreatinemic compared to all other groups (12, 30) and baseline plasma levels of NEFA and TG were also highest in LOW-HCHF sheep. However, except for TG, as described later, we did not find any modules that correlated with plasma levels of these metabolites. This suggests that other metabolically active organs, such as the liver and kidney, may have played more significant roles in developing these adverse metabolic traits.

We have previously identified aurora kinase A (*AURKA*), integrin subunit beta 2 (*ITGB2*), cathepsin S (*CTSS*), and transmembrane immune signaling adaptor (*TYROBP*) as hub genes in PER, whose expression levels were altered in adult sheep with different histories of pre- and/or early postnatal nutrition (10). Using WGCNA, we confirmed *AURKA*, *ITGB2*, *CTSS*, and *TYROBP* as hub genes, which were identified in the coexpressed modules in PER♂, whereas *AURKA* was identified as a hub gene in PER♀, and these hub genes have been associated with obesity comorbidities (86–88).

Primary cilia are microtubule-based organelles protruding from the cell, and they play an important role in cell-cell interactions and signaling. They are tightly associated with the cell cycle progression and hence crucial for cell growth and development (89–91). The ciliary disassembly regulators such as *AURKA*, polo like kinase 1 (*PLK1*), polo like kinase 4 (*PLK4*), kinesin family member 2 A (*KIF2A*), and kinesin family member 2 A (*KIF24*) expression levels are associated with shortened and deficient cilia (86). This might lead to defective adipogenesis and adipocyte hypertrophy (86) and even promote the formation of tumors (87). The present study identified *AURKA* as a hub gene in PER tissue in both sexes but not in SUB.

A growing number of studies have identified *AURKA* as a key player in different types of cancers, such as gastrointestinal and pancreatic cancers, and it has been proposed as a molecular therapeutic target for tumorigenesis (92, 93). Although several preclinical studies supported *AURKA* as a therapeutic target for cancers (92, 93), further studies are required to establish the role of *AURKA* in adipose development and its implication for obesity using an animal model on a large scale and in humans. A better understanding of *AURKA* activity in adipose tissue development can provide insight into the prevention and treatment of adipose dysfunction-associated diseases, and potential drugs targeting *AURKA* could be designed for tests in clinical trials for future treatment.

Studies in animal models and humans have demonstrated that *CTSS*, *ITGB2*, and *TYROBP* are markers for obesity and represent possible molecular links between obesity and obesity comorbidities, such as cardiovascular diseases and atherosclerosis (94–100). In addition, *ITGB2* and *TYROBP* genes’ expression levels correlate with blood levels of low-density lipoprotein cholesterol (LDL-C) in the mini-pig atherosclerosis model (94). In contrast, specific variants of *CTSS* were associated with metabolic risk factors for CAD, including plasma levels of lipoprotein A1 and high-density lipoprotein cholesterol (HDL-C) (99) and serum total cholesterol level (100). Furthermore, the haplotype analysis of rs16827671 and rs11576175 showed combined effects on hypertriglyceridemia and hypercholesterolemia (100).

In atherosclerosis, the LDL-C is known to stimulate innate and adaptive immunity (101, 102), in which macrophages and the ingestion of lipids cause foam cell formation that drives the development in the arterial wall of atherosclerosis plaques composed of foam cells, calcium, lipids, and other components (103). Interestingly, these hub genes (*CTSS*, *ITGB2*, and *TYROBP*) were identified in one of the coexpressed modules in PER♂ (but not PER♀), which showed a positive correlation with the basal plasma levels of TG, which may suggest a link between PER, immunity, and plasma level of TG in males.

### Conclusions

Our study demonstrated depot-specific and sexually dimorphic molecular alterations in response to early life malnutrition programming that might underpin changes in the observed expandability traits of adipose tissues later in life (adulthood). Particularly prenatal undernutrition had long-term implications for fundamental developmental and functional biological processes in PER in adulthood through programming fundamental molecular mechanisms such as regulatory factors governing overall gene expression and the cell cycle and hence tissue development and differentiation. Changes in the gene expression and epigenetic regulators, such as chromatin and H3-K27 trimethylation in PER♂ and PER♀ gene networks, respectively, may account for reduced and increased hypertrophic capacity by PER adipocytes in LOW♂ and LOW♀. In addition, specific to the PER♀ gene network, prenatal malnutrition targeted mitochondria and OXPHOS-related functions, which were significantly associated with increased numbers of hypertrophied (large) adipocytes in LOW♀. In SUB (and only in males), the only modules correlated with prenatal nutrition were networks functionally related to ADP metabolism and development (e.g., tube closure and mammary gland). Altogether, this shows that PER in both sexes was the primary target of prenatal malnutrition, with long-term implications for gene expression patterns possibly mediated in part by epigenetic modifications.

Nevertheless, we found no evidence to suggest that neither PER nor SUB should have played any particular role in the development of persistent hypercholesterolemia, hyperuricemia, or hypercreatinemia observed in the adult LOW-HCHF sheep. *AURKA* was identified as the hub gene in the coexpressed module of PER in both sexes, especially in PER♀ to be affected by exposure to especially prenatal (under-)nutrition, causing long-term persistent changes in PER development. Further studies are needed to better understand the role of *AURKA* in third-trimester programming of adipose tissue development to provide an insight into the possibilities for preventing the long-term risks for adipose dysfunction-associated diseases and potential treatments targeting *AURKA.*

## DATA AVAILABILITY

The RNA-Seq data analyzed, discussed, and presented in this study were deposited in National Center for Biotechnology Information’s Gene Expression Omnibus (GEO) and are accessible through GEO series accession number GSE166662 through the following link: https://www.ncbi.nlm.nih.gov/geo/query/acc.cgi?acc=GSE166662.

## SUPPLEMENTAL MATERIAL

10.6084/m9.figshare.20493921 Supplemental Figs. S1 and S2 and Supplemental Tables S1–S4: https://doi.org/10.6084/m9.figshare.20493921.

## GRANTS

This study was part of the research program of the Center for Foetal Programming (CFP), Denmark, and animal experiments were funded by the Danish Council for Strategic Research, Denmark (Grant 09067124). The Ministry of Higher Education, Malaysia, funded the RNA-sequencing analyses. The Ministry of Higher Education, Malaysia, and the Universiti Putra Malaysia sponsored the main authors’ Ph.D. scholarship.

## DISCLOSURES

No conflicts of interest, financial or otherwise, are declared by the authors.

## AUTHOR CONTRIBUTIONS

M.O.N. conceived and designed research; M.O.N. performed experiments; S.A., M.H.D., S.M.S. and Z.C. analyzed data; S.A. and M.O.N. interpreted results of experiments; S.A. prepared figures; S.A. drafted manuscript; S.A., M.H.D., S.M.S., Z.C., and M.O.N. edited and revised manuscript; S.A., M.H.D., S.M.S., Z.C., and M.O.N. approved final version of manuscript.
